# Leptomeningeal carcinomatosis mimicking Creutzfeldt–Jakob disease: clinical features, laboratory tests, MRI images, EEG findings in an autopsy-proven case

**DOI:** 10.1007/s10072-012-1021-1

**Published:** 2012-04-17

**Authors:** Margherita Francolini, Luigi Sicurella, Nicolò Rizzuto

**Affiliations:** Division of Neurology, S. Antonio Abate Hospital, Trapani, Italy

**Keywords:** Cerebrospinal fluid, Leptomeningeal carcinomatosis, Creutzfeldt–Jackob disease

## Abstract

Leptomeningeal carcinomatosis (LC) refers to diffuse seeding of the leptomeninges by tumor metastases. The clinical presentation may differ and the diagnosis may be difficult especially when cancer has not yet been diagnosed. We report a case of LC, where the clinical picture and a specific change in cerebrospinal fluid suggested the diagnosis of a prion disease.

## Background

Leptomeningeal carcinomatosis (LC) refers to diffuse seeding of the leptomeninges by tumor metastases. This condition was first reported in 1870 by Eberth, although this term was commonly used only in the early 20th century. LC occurs in an estimated 20 % of patients diagnosed with cancer; in adults, it is most frequently found in breast and lung carcinoma as well as in melanoma. In a few cases, prostate cancer also can spread to the leptomeninges. In children, hematogenous malignancies and primitive neuroectodermal tumors are the most common cause of leptomeningeal carcinomatosis, but pilocytic astrocytomas (which are generally low grade) occasionally can demonstrate this type of aggressive behavior [1, 2]. Patients typically present with symptoms caused by the effects of tumor emboli on subarachnoid nerve roots, direct invasion into the spinal cord or the brain, or cerebrospinal fluid (CSF) obstruction. Signs and symptoms referable to one or several cranial or spinal nerve roots are the typical presentation of leptomeningeal carcinomatosis (such condition), associated soon with headache, confusion and other type of neurological involvement [3, 4]. However, the clinical presentation may be different and the diagnosis is difficult, especially in the cases where the diagnosis of cancer is lacking (Table 1).

**Table 1:** Incidence of clinical symptoms and signs of leptomeningeal carcinomatosis [3, 4]

Clinical features	Frequency (%)
Cranial nerve palsies (any)	75
Cerebral signs	66
Headache	66
Spinal nerves	60
Mental changes	45
Limb weakness	44
Difficulty walking	33
Meningism	21
Sensory abnormalities	21
Nausea–vomiting	20
Cerebellal signs	16
Fits	12
Dizziness	9
Autonomic dysfunction	

The outlook is grim; untreated patients are unlikely to survive more than 4–6 weeks. Intrathecal chemotherapy and/or radiation can increase the survival to some extent, depending partly on the cell type of the involved tumor, but most patients succumb to their disease within 6–8 months [1, 2, 3–13].

We report a case of LC, the clinical picture of which associated with a peculiar change of CSF suggested in vita the diagnosis of a prion disease.

## Case report

**Fig. 1:**
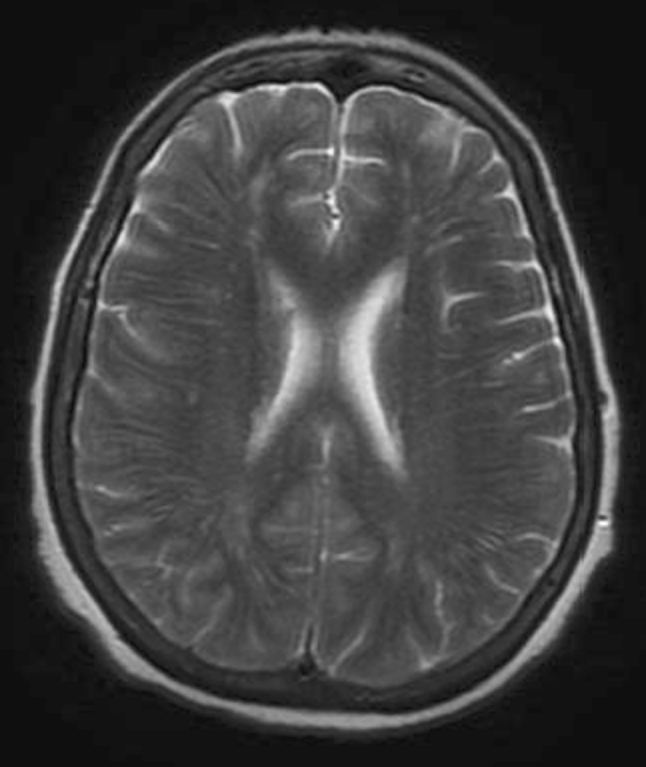
MRI (GE-Philips 1.5T) Patient’s axial T2 weighted normal image

**Fig. 2:**
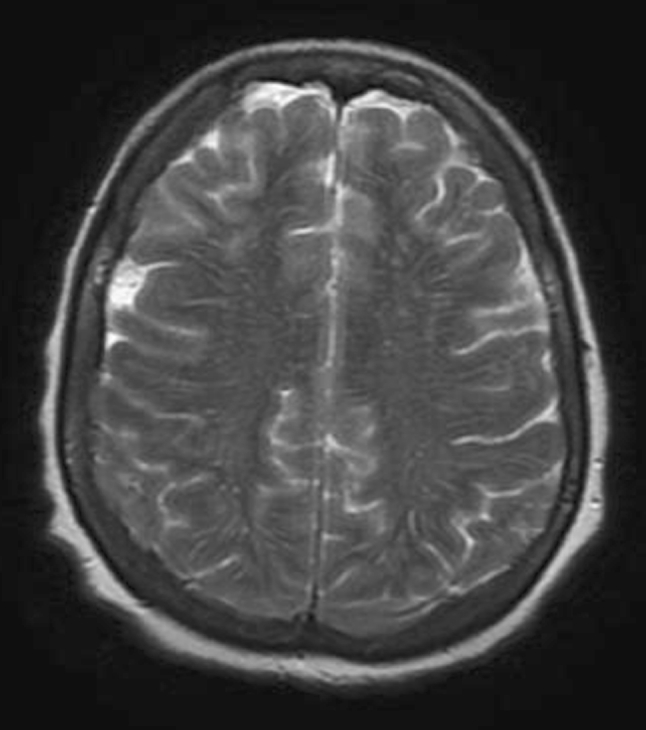
MRI (GE-Philips 1.5T) Patient’s axial T2 weighted image (*top of the head*: absence of meningeal abnormalities)

A 56-year-old woman affected by depression and obsessive compulsive traits, migraine and fibromyalgia for years came to our observation because of headache and marked thymic deflection, inability to work and weight loss. She had been admitted to a private clinic and subjected to brain MRI that did not show any abnormal findings (Figs. 1, 2); she was discharged after 10 days with the diagnosis of depressed mood. Despite the drug therapy, the symptoms worsened and became associated with impoverishment of language, confusion, and severe postural instability. The patient was then hospitalized in the neurological department of the city hospital.

On admission, the clinical picture was mainly characterized by a severe cognitive decline, dysarthria, ataxic gait, plastic hypertonia and postural instability.

Blood tests showed elevated IES (45 mm), a slight increase of CEA (4.9 ng/ml). VDRL/TPHA and HIV tests were negative; total body TC did not reveal any evidences of inflammatory, vascular or neoplastic processes, mainly neither abdominal nor pulmonary cancer.

The electroencephalogram showed a marked slowing of background activity, but neither paroxistic activities (P, PO, PPO) nor periodic sharp wave complexes were found. Visual evoked potential (VEP) were markedly altered bilaterally.

The patient was again subjected to brain MRI with contrast showed no abnormalities of the brain parenchyma (Figs. 1, 2).

The clinical condition impaired rapidly within the following days; confusion and disturbances of alertness were rapidly substituted by stupor and coma, but she never showed tremor or seizure.

The CSF collected by lumbar puncture appeared clear; the chemical and physical examination demonstrated a slight increase of proteins (57.5 mg/dl) with reduction of glucose (13 mg/dl). No cells were detected and cytology did not show any atypical cells. CSF was positive for 14-3-3 protein and a content of Tau protein of >4,000 pg/ml.

In conclusion, the data available to us were the following: a rapid cognitive decline with cerebellar and extrapyramidal signs, akinetic mutism, MRI negative for parenchymal abnormalities and positivity of CSF for 14-3-3 and Tau proteins.

The 14-3-3 protein is present in many inflammatory, degenerative and paraneoplastic diseases; when there is the clinical suspicion of prion disease, the presence of the 14-3-3 protein associated with Tau protein in excess of 1,000 pg/ml, assumes a positive diagnostic-predictive significance in 97 % of patients. For this reason, we made the diagnosis of “Probable Creutzfeldt–Jakob disease” in accordance with the latest diagnostic criteria.

On 9 December, after 26 days of hospitalization, the patient dies.

Histological examination performed on the left cerebral hemisphere.

### Macroscopic description

The cerebral hemispheres have a normal configuration. The leptomeninges appear smooth and transparent. The grooves are slightly dilated and the cortical convolutions are slightly atrophic. No significant areas of softening. There are no signs of huncus or cingulate gyrus’s herniation. The blood vessels examined show a normal configuration and mild atherosclerosis. After coronal resection of the cerebral hemisphere, the cortex appears macroscopically intact.The ventricular system is not deformed or dilated. The semioval center is normal. The hippocampus, amygdala, basal ganglia and cerebellum appear normal. The locus coeruleus and substantia nigra have normal pigmentation.

### Microscopic examination

In all sections of the cerebral cortex, hippocampus and cerebellum cellular infiltration in neoplastic nature that affects the meninges is observed. The infiltrate consists of cells with moderate nuclear pleomorphism, medium size, with clearly visible cytoplasm, polygonal shape or cylindrical, often arranged in nests aspect of “epithelial”. The infiltration of tumor cells is almost restricted to meningeal spaces.

### Immunohistochemical examinations

Immunohistochemical investigations were carried out using antibodies specific for the following proteins: GFAP, CKWS (broad-spectrum cytokeratins) and Ki67.

The neoplastic cells were GFAP negative and CKWS positive. Ki67 staining showed a positive mitotic index in approximately 5 % of neoplastic cells.

### Molecular studies

The Pursuit of pathological prion protein (WB) gave negative results.

### Diagnosis

Histological examination performed on the left cerebral hemisphere showed an histology of neoplastic meningeal infiltration of epithelial nature.

## Discussion

Meningeal carcinomatosis is a rare complication that occurs in 20 % of cancer patients many of whom suffering from lung or breast cancer [3, 4, 14–16]. The clinical presentation is heterogeneous and in many cases, that is difficult to recognize as expression of meningeal pathology: the diagnosis is based on the involvement of one or more cranial nerve associated with headache and confusion, due to raised intracranial pressure and is sustained by the demonstration of malignant cells in the CSF.

The diagnostic management of LC is based on the CSF examination with cytology and biochemical analysis as well as on the radiological investigation, mainly with MRI [10].

Basically, CSF examination includes the fluid cytology, estimation of the opening pressure, as well as measurement of protein and glucose concentrations. The measurement of other biochemical markers is less reliable. Lumbar puncture for CSF cytology remains the standard diagnostic procedure. The demonstration of malignant cells in the CSF is still the cornerstone of the final diagnosis. An adequate sample of 5 ml or more increases the percentage of positive examination. A positive CSF cytology is found on the initial lumbar puncture in 50–70 % and in nearly all cases after three attempts. False-positive cytologies are associated with infectious or inflammatory diseases demonstrating reactive lymphocytes. Increased CSF opening pressure is found in 50–70 % of the patients and depends on the extent of the leptomeningeal involvement. Elevated CSF protein and low glucose are observed in approximately 75 and 40 % of the cases, respectively. In our case, the cytology was negative both in the first and the second CSF prelevament.

MRI and CT demonstrate multiple masses within the subarachnoid space, hydrocephalus without a discernible cause, or diffuse leptomeningeal enhancement [1, 2, 10–12]. The latter enhancement pattern has been referred to as cake icing or zuckerguss (German for sugar icing) and can be found in the brain, spine, or both, as shown in the figures. As the diagnostic accuracy of lumbar puncture is only 50–60 % after a single LP and 90 % after three LPs, MRI is considered complementary and can be invaluable, detecting up to 50 % of cases with false-negative LPs [1, 2, 4, 8].

In our case, neither CSF abnormalities nor MRI alteration addressed us to the diagnosis of MC; on the contrary, the clinical presentation with rapidly progressing cognitive failure preceded by cerebellar and extrapyramidal signs suggested the diagnosis of Creutzfeldt–Jakob disease mainly by the demonstration of markers such as 14-3-3 and Tau proteins. The diagnostic procedure was correct even though the diagnosis was wrong and other most sophisticated diagnostic methods, except the brain biopsy, would not achieved the diagnosis. We did not find the principal tumour although the patient has been submitted to neuroimaging and CSF examinations. The most likely hypothesis could be that the primary tumour was a misunderstood malignant melanoma, confirmed by the negativity of total body CT scan. In this case report, only brain biopsy addressed the correct diagnosis.

Due to the peculiar features that characterize the clinical picture and the dramatic evolution, we think this case is worthy to be published as short case report.
